# Recovery of the 20 Hz Rebound to Tactile and Proprioceptive Stimulation after Stroke

**DOI:** 10.1155/2018/7395798

**Published:** 2018-02-28

**Authors:** Eeva Parkkonen, Kristina Laaksonen, Lauri Parkkonen, Nina Forss

**Affiliations:** ^1^Department of Neuroscience and Biomedical Engineering, Aalto University School of Science, Espoo, Finland; ^2^Aalto Neuroimaging MEG-Core, Aalto University School of Science, Espoo, Finland; ^3^Department of Neurology, Helsinki University Hospital and Clinical Neurosciences, Neurology, University of Helsinki, Helsinki, Finland

## Abstract

Sensorimotor integration is closely linked to changes in motor-cortical excitability, observable in the modulation of the 20 Hz rhythm. After somatosensory stimulation, the rhythm transiently increases as a rebound that reflects motor-cortex inhibition. Stroke-induced alterations in afferent input likely affect motor-cortex excitability and motor recovery. To study the role of somatosensory afferents in motor-cortex excitability after stroke, we employed magnetoencephalographic recordings (MEG) at 1–7 days, one month, and 12 months in 23 patients with stroke in the middle cerebral artery territory and 22 healthy controls. The modulation of the 20 Hz motor-cortical rhythm was evaluated to two different somatosensory stimuli, tactile stimulation, and passive movement of the index fingers. The rebound strengths to both stimuli were diminished in the acute phase compared to the controls and increased significantly during the first month after stroke. However, only the rebound amplitudes to tactile stimuli fully recovered within the follow-up period. The rebound strengths in the affected hemisphere to both stimuli correlated strongly with the clinical scores across the follow-up. The results show that changes in the 20 Hz rebound to both stimuli behave similarly and occur predominantly during the first month. The 20 Hz rebound is a potential marker for predicting motor recovery after stroke.

## 1. Introduction

Around 80% of all acute stroke patients suffer from upper limb paresis hampering daily activities [1, 2]. At present, rehabilitation after stroke is mainly based on symptoms of the patients rather than on underlying neurophysiological changes. A better understanding of stroke-induced changes in brain functions is required to develop more individually tailored and more efficient rehabilitation.

Integration of somatosensory feedback with motor output is essential for fluent motor performance, and it is tightly coupled with changes in cortical excitability; afferent somatosensory input is known to alter motor-cortex inhibition [3–9]. Motor-cortex excitability is reflected in the modulation of the 20 Hz rhythm; activation of the motor cortex suppresses this rhythm whereas the subsequent rebound after movement cessation reflects inhibition or deactivation of the motor cortex [6, 10–13]. Both animal and human studies have shown that an acute stroke induces changes in motor-cortex excitability [14–18]. Our previous MEG studies in stroke patients using tactile [19] and proprioceptive [20] stimulation suggest that alterations in motor-cortex excitability after stroke are probably due to both changes in local excitatory–inhibitory circuits and disturbed afferent input, which lead to impaired sensorimotor integration. To further understand the mechanisms affecting motor-cortex excitability and recovery after stroke, we compared how two different types of afferent input modulate motor-cortex excitability during one-year recovery from stroke.

We employed magnetoencephalography (MEG) to compare the effect of tactile and proprioceptive stimulation of the index fingers on the 20 Hz rhythm at one week, one month, and one year after stroke and thereafter correlated the results with clinical recovery of the patients.

## 2. Methods

The data of the control subjects and the passive movement-induced changes in the 20 Hz rhythm in the patients are obtained from our previous two studies [20, 21]. Modulation of the 20 Hz rhythm to tactile stimuli, presented here for the first time, was recorded in the same sessions as passive movement data.

### 2.1. Subjects

Thirty patients with first-ever stroke in the territory of the middle cerebral artery and related unilateral upper limb paresis were recruited from the Department of Neurology, Helsinki University Hospital (HUH). Clinical neurological examination was performed at the time of recruitment to include patients with hand weakness or clumsiness. Patients with earlier neurological diseases, mental disorders, prior neurosurgical operations, or unstable cardiovascular/general condition were not included. Seven patients were excluded later during follow-up; two died, four declined the second or third MEG recording, and the data of one patient were contaminated with artifacts preventing reliable analyses. Eventually, 23 patients participated the entire study (10 females, age 45–78 years, mean 65 ± 2 years; Table 1). The control group comprised 22 healthy subjects (11 females, age 42–72 years, mean 59 ± 2.0 years). The Local Ethics Committee of the Helsinki and Uusimaa Hospital District approved the study protocol, and all subjects assigned written informed consent prior to the measurements.

### 2.2. Clinical Evaluation

NIHSS (National Institutes of Health Stroke Scale; Table 1) evaluation, hand motor function, tactile sensitivity, and proprioception were assessed in conjunction with the MEG recordings 1–7 days (T_0_), 1 month (T_1_), and 12 months (T_2_) after stroke. According to NIHSS, stroke impairment can be classified as mild (NIHSS < 8), moderate (NIHSS 8–16), and severe (NIHSS > 17). An occupational therapist tested the manual dexterity of both the impaired and healthy hands of the patients by using the Box-and-Block test (BB; number of cubes moved from one compartment to another in 60 s; Table 2). The tactile detection threshold was evaluated with von Frey Filaments (20 filaments; 3.22–3.61 normal/reduced light touch; 3.84–4.31 reduced protective sensation; 4.56–6.65 no protective sensation; 6.65 no measurable tactile sense; Table 2). A qualitative test was used for evaluation of proprioception; the impaired hand was placed to different positions, and the patient reproduced the positions without seeing the healthy hand; the ability to mimic the positions with the healthy hand was evaluated to be normal or abnormal. The qualitative test showed that proprioceptive sense of the impaired hand was normal only in 5/23 at T_0_, in 8/23 at T_1_, and in 11/23 at T_2_.

### 2.3. MEG Recordings and Neuroradiological Evaluations

A whole-scalp MEG system (306 channels; 204 planar gradiometers and 102 magnetometers; Vectorview™; Elekta Oy, Helsinki, Finland) was employed for the recordings. The measurements of 18 control subjects were performed in Aalto University and four controls and all patients with similar devices in the BioMag Laboratory (HUH, Finland). During the recordings, the subjects were either in a sitting or supine (four patients at T_0_) position and instructed not to pay attention to the finger lift or tactile stimulation, to relax, and to avoid excessive blinking.

Four indicator coils as well as three anatomical landmarks (right and left preauricular points and nasion) and 50–100 additional points on the head surface were used for coregistration. The MEG and vertical electrooculogram signals were pass-band filtered to 0.03–330 Hz and digitized at 1000 Hz. About 60 averaged trials were accepted for each hand while acquiring continuous data for analysis. In addition, resting state data with eyes open and eyes closed (3 min each) were recorded.

To determine the lesion site and size, anatomical magnetic resonance images (MRIs) were taken at T_0_ and T_1_ with a 3T MRI scanner (Philips Achieva 3T, Philips Medical Systems, Best, The Netherlands). The MRIs revealed two patients with cortical, 15 with cortico-subcortical, and six with subcortical infarcts; of which, 16 patients had right and seven left hemispheric lesions. The size of the lesion varied from 0.24 to 218.5 cm^3^ (mean 40 ± 12 cm^3^; Table 1).

### 2.4. Stimulation

#### 2.4.1. Passive Movement

The index finger was lifted briskly by a laboratory nurse once every 3 s (in the patients, first the healthy and then the impaired side) with a rigid aluminum stick attached with a Velcro strap to the phalanx. Cutaneous tactile stimulation was minimized by covering the middle phalanx with a surgical tape and by ensuring that the fingertip did not touch the device during the movement. A 3-axis accelerometer (ADXL335 iMEMS accelerometer Analog Devices Inc., Norwood, MA, USA) linked to the MEG system was attached on the nail of the index finger to determine the finger kinematics. Reliable accelerometer signals were acquired in 17 controls and 16 patients. The average lag time (time from actual onset of passive finger movement to recorded movement onset) was calculated and used for the subjects with no accelerometer signals.

The rhythm and amplitude of the movements were kept constant by monitoring the moving index finger with two optical gates (lower and upper) separated by 30 mm along the direction of the movement; only movements passing through both gates within 500 ms were accepted as valid trials for on-line averaging and later for off-line analysis, ~60 for each hand.

In the patients, the peak acceleration of the index finger did not differ significantly between the healthy and the impaired hand at any time point. Neither were differences within one hand observed between T_0_ and T_1_. However, passive movements of both the healthy and impaired hands in the patients were brisker at T_2_ than at T_0_ (*p* < 0.001) and at T_1_ (*p* < 0.01). In the controls, the peak acceleration of the passive movement did not differ between the right and left index fingers. At T_2_, the peak acceleration in the patients for both the healthy and impaired hands was brisker than in the controls (*p* < 0.001) but no significant differences between patients and controls were found at T_0_ or T_1_. The movement duration was significantly shorter (*p* < 0.01) in the patients versus controls in all measurement sessions. However, the movement duration of either hand of the patients did not differ between T_0_ and T_1_ [20].

#### 2.4.2. Tactile Stimulation

Pneumatic diaphragms driven by compressed air were used to deliver tactile stimuli (duration 140 ms, peak at 50 ms) to the tips of the index fingers alternately with an interstimulus interval (ISI) of 1.5 s (3 s for one side). Around 60 on-line-accepted trials were collected for each finger for later off-line analysis.

More detailed description of tactile and proprioceptive stimulation is presented in our previous study in healthy controls [21].

### 2.5. Data Analysis

Temporal signal-space separation method (tSSS) [22] was used to suppress environmental magnetic interference from the MEG data. Head movements were compensated with the MaxFilter software (version 2.2.11; Elekta Oy) [23, 24]. Only data from the 204 planar gradiometer channels were used for subsequent analysis.

To determine the peak amplitudes and frequencies of spontaneous brain activity, the amplitude spectra were estimated from the resting-state data (eyes open) with the Welch method using 2048-sample Hanning-windowed segments. The strongest peaks were found in the 15–25 Hz range in both the controls and the patients; this band was chosen for further analysis in all subjects. The strength of *β*_1_- and *β*_2_-peaks (9–15 and 8–11 fT/cm, resp.) did not differ significantly between the hemispheres, between time points, or between patients and controls. Time-frequency representations (TFR) of passive movement and tactile stimulation responses were calculated over all channels for the 3–40 Hz range with 7-cycle Morlet wavelets, to visually assure the frequency range of the strongest modulation.

The temporal spectral evolution method (TSE) [13] was used to quantify the modulation of the 20 Hz rhythm; the continuous data were first filtered to 15–25 Hz, rectified, and averaged (−100–3000 ms) time-locked to stimulus onset.

Peak amplitudes of suppression and rebound over the sensorimotor cortex were quantified in both the ipsi- and contralateral hemispheres with respect to the moved/stimulated hand from four channels (two from each hemisphere) showing the strongest suppression/rebound of 20 Hz activity. The relative peak amplitudes were calculated as percentage of amplitude changes with respect to the individual prestimulus baseline (−100–0 ms).

### 2.6. Statistical Analysis

The normality of the data was tested with the Kolmogorov–Smirnov (KS) test; with four variables, the null hypothesis of a normal distribution could be rejected at *p* < 0.05. To ensure that all variables are normally distributed, we converted the original values *x* into new values *y* = ln(*x* + 1) where ln(·) is the natural logarithm. After this transformation, the KS test indicated normal distribution of all variables. These transformed variables were used for statistical analyses.

The kinematics of passive movements and clinical test results in the patients between the impaired and healthy hands were compared with a two-way (hand: impaired and healthy; time: T_0_, T_1_, and T_2_) repeated measures ANOVA. The kinematics of passive movements were compared between the patients and the controls (right and left hands pooled) with one-way, six-level (2 × hand; 3 × time) ANOVA [20].

The TSE results from all sessions (T_0_, T_1_, and T_2_) were evaluated in both the affected (AH) and unaffected hemispheres (UH) to both impaired and healthy hand tactile stimulation and passive movement. The variance within factor time, hemispheres (AH/UH), and side of stimulation was analyzed with a two-way within-subject ANOVA. Significant (threshold *p* < 0.05) main effects (F) were compared with paired sample *t*-tests. Independent sample *t*-tests were used when comparing effects between controls and patients.

As rebound amplitudes were clearly larger to passive than to tactile stimuli even in the healthy subjects, direct comparison of amplitudes was not possible. To compare the recovery rates of the rebounds to the two stimulus types, the relative rebounds were normalized with respect to the relative rebound of the healthy hand in the unaffected hemisphere at T_2_. Likewise, to compare the recovery of the hand motor performance (BB test and tactile sense), the clinical scores of the impaired hand were normalized with those of the healthy hand at T_2_.

Spearman's nonparametric correlation was applied to test for associations between the lesion volumes and clinical variables (scores of BB test, tactile sense) and MEG responses (threshold *p* < 0.05).

## 3. Results

### 3.1. NIHSS

According to the NIHSS evaluation, the severity of the impairment caused by stroke varied from mild to moderate; NIHSS < 17 in all the patients (Table 1). Note that NIHSS was zero in three patients in the acute phase despite of their upper limb paresis. This is due to evaluation of upper arm strength in NIHSS; zero point is obtained if the patient is capable of lifting the arm and holding it up for 10 seconds despite total lack of distal hand movements.

### 3.2. Tactile Sense

**Figure 1:**
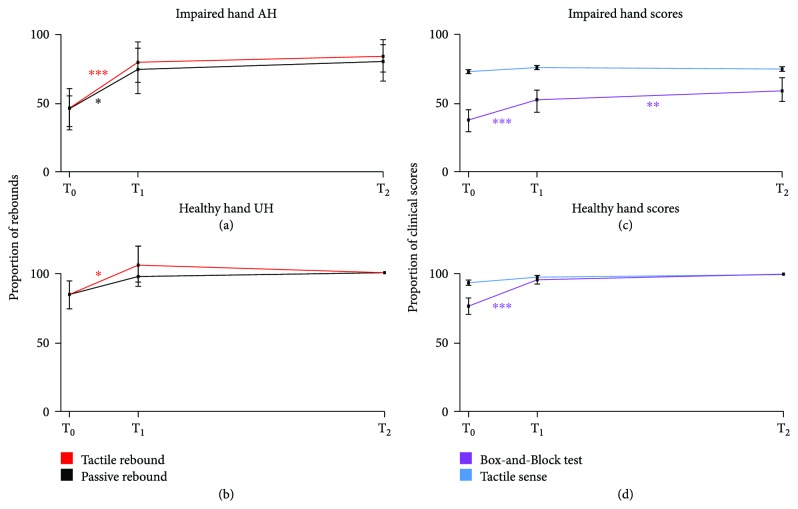
(a, b) Recovery rate of the relative rebounds in the affected hemisphere of the patients to tactile stimulation versus passive movement of the impaired hand (a) and in the unaffected hemisphere to tactile versus passive stimulation of the healthy hand (b), both normalized to the corresponding rebound in the unaffected hemisphere at T_2_. (c, d) Recovery rate of the Box-and-Block scores and tactile sensitivity of the impaired (c) and healthy (d) hands of the patients normalized to the corresponding clinical scores at T_2_. AH = affected hemisphere; UH = unaffected hemisphere; T_0_ = 1–7 days; T_1_ = 1 month; T_2_ = 12 months after stroke; and BB = Box-and-Block test. ^∗^*p* < 0.05, ^∗∗^*p* < 0.01, and ^∗∗∗^*p* < 0.001.

At T_0_, tactile sensitivity of the impaired hand was significantly diminished (4.56 ± 0.22 versus 3.74 ± 0.08; *p* < 0.01) compared to that of the healthy hand (Table 2). Tactile sensitivity of the impaired hand improved significantly from T_0_ to T_1_ (*p* < 0.05) but not from T_1_ to T_2_ and remained significantly weaker compared to that of the healthy hand (*p* < 0.05). In the healthy hand, tactile sensitivity improved significantly from T_0_ to T_2_ (3.74 ± 0.08 versus 3.57 ± 0.04; *p* < 0.05) but not from T_0_ to T_1_ or T_1_ to T_2_.  Figure 1 shows how tactile sensitivity of the impaired and healthy hands recovered during the one-year follow-up.

### 3.3. Hand Motor Performance

The results of the BB test of the impaired and healthy hands have been presented in our previous study [20], and they are shown here in Table 2. During the one-year follow-up, BB of the impaired hand improved from T_0_ to T_1_ (*p* < 0.001) and from T_1_ to T_2_ (*p* < 0.01). However, at all time points, BB scores of the impaired hand were significantly worse (*p* < 0.001) than those of the healthy hand. BB improved also for the healthy hand from T_0_ to T_1_ (*p* < 0.001) but not significantly from T_1_ to T_2_.  Figure 1 shows the recovery of BB scores of the impaired and healthy hands (normalized to the scores of the healthy hand at T_2_) during the one-year follow-up.

At T_0_ and T_1_, BB scores of the impaired hand were lower than the values of a healthy population (matched for gender, age, and the side of the tested hand) [25] in all patients and at T_2_ in 21/23 patients. The healthy hand BB scores were lower than those of the healthy population in 21 patients at T_0_, 19 at T_1_, and 15 at T_2_.

### 3.4. Modulation of the ~20 Hz Rhythm

#### 3.4.1. Peak Latencies of Suppression and Rebound

The baseline levels of the 20 Hz rhythm in the patients and between the patients and controls did not differ significantly between the hemispheres or between different time points. In the patients, the suppression of the 20 Hz rhythm peaked at 530 ± 10 ms after passive movement and at 270 ± 10 ms after tactile stimulation; the subsequent rebound peaked at 1370 ± 30 ms and at 690 ± 20 ms, respectively. In the controls, the suppression peaked at 540 ± 10 ms after passive movement and at 300 ± 10 ms after tactile stimulation and the rebound at 1450 ± 30 ms and 790 ± 10, respectively. No differences in peak latencies were detected between the hemispheres or between the patients and control subjects. In all measurements, the peak latencies of suppression and rebound to passive movement were significantly longer (*p* < 0.001) compared to those to tactile stimulation.

No significant differences were detected in the strength of the suppression between the hemispheres of the patients, between different time points, or between the patients and the controls.

#### 3.4.2. 20 Hz Rebound Strength to Tactile versus Proprioceptive Stimulation

The maximal 20 Hz rebounds both to passive movement and to tactile stimulation were detected over the same planar gradiometer channels as the strongest beta peaks in the amplitude spectra of the resting-state data; the location of the maximal rebound was found over the rolandic area anterior to that of the maximal suppression.

Both tactile and proprioceptive stimulation modulated bilaterally the 20 Hz rhythm, but the effect was much stronger in the contralateral hemisphere to the stimulated hand, in line with earlier findings [12, 13, 19–21]. Therefore, in the present study, we compared the rebounds of the hemisphere contralateral to the stimulated hand. The rebound strengths (mean ± SEM) of the patients and controls are presented in Table 3.

**Figure 2:**
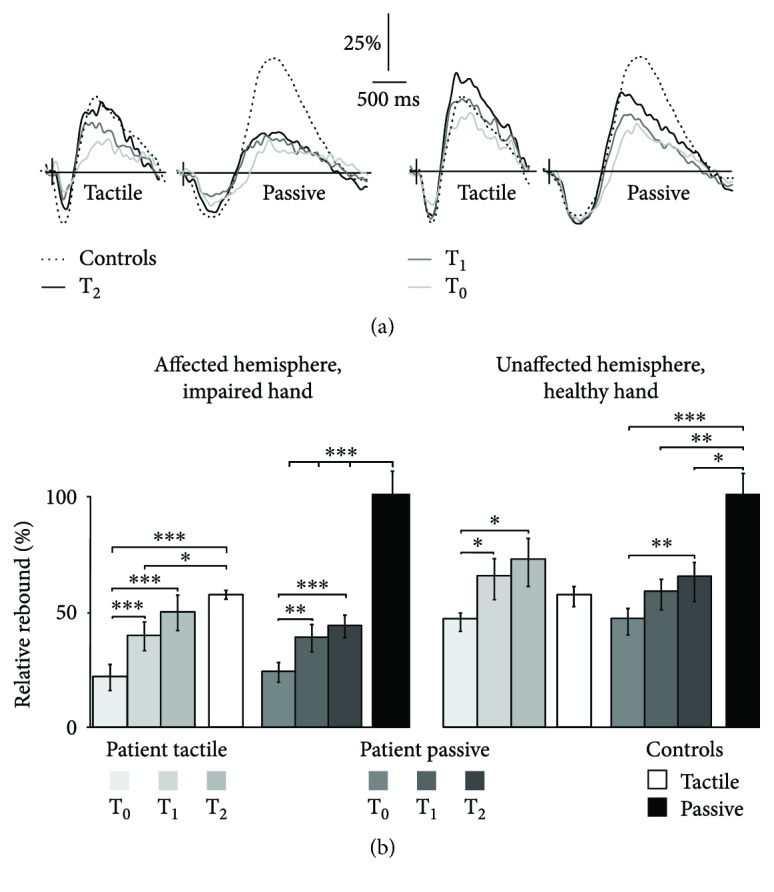
Modulation of the ~20 Hz rhythm to tactile stimulation and passive movement. (a) Grand average TSE of the ~20 Hz rhythm: rebound strengths (% with respect to the prestimulus baseline) in the affected and unaffected hemispheres to tactile stimulation and passive movement of the impaired and healthy hands (contralateral stimulation) at T_0_ –T_2_ in the stroke patients (*N* = 23) and to contralateral stimulation in the controls (hemispheres pooled, hence *N* = 44). (b) Rebound strengths (% with respect to the prestimulus baseline) in the affected and unaffected hemispheres to tactile stimulation and passive movement of the impaired and healthy hands in the patients and to contralateral stimulation in the controls (hemispheres pooled). T_0_ (1–7 days), T_1_ (1 month), and T_2_ (12 months) after stroke. ^∗^*p* < 0.05, ^∗∗^*p* < 0.01, and ^∗∗∗^*p* < 0.001.

Figure 2(a) shows the grand average TSE of the 20 Hz band in the affected and unaffected hemispheres to contralateral tactile stimulation and passive movement; in each patient, the channel showing the maximal rebound was selected, and the TSEs of these channels were then averaged and divided by the mean baseline value. In the controls, no differences between the rebound strengths within one stimulus type between the left and right hemispheres were detected; hence, the rebounds in both hemispheres to contralateral stimuli were pooled [21].

#### 3.4.3. Affected Hemisphere, Impaired Hand Stimulation

Figure 2(b) shows the relative rebound (% of the baseline) strengths to tactile stimulation and passive movement in the patients during the 12-month follow-up period. Rebounds to tactile stimulation were identified in 13/23 patients at T_0_. At T_1_ and T_2_, all the patients showed reliable rebounds. To passive movement, measurable rebounds were found in 17 patients at T_0_, in 21 at T_1_, and in all 23 patients at T_2_.

The rebound strength to tactile stimulation increased significantly from T_0_ to T_1_ and T_2_ (*p* < 0.001), but no significant increase was detected from T_1_ to T_2_. Accordingly, the rebound to passive movement increased significantly from T_0_ to T_1_ and to T_2_ (*p* < 0.01 and *p* < 0.001, resp.) but not from T_1_ to T_2_. The rebound strength to tactile stimulation reached the level of the controls by T_2_ whereas the rebound strength to passive movement remained significantly (*p* < 0.001) weaker than that of the controls at T_2_ (46% of the rebound of the controls).

#### 3.4.4. Unaffected Hemisphere, Healthy Hand Stimulation

The rebounds to both stimuli were identified in all patients at all time points. Figure 2(b) shows that the rebound strength to tactile stimulation increased significantly from T_0_ to T_1_ and to T_2_ (*p* < 0.05) but not from T_1_ to T_2_. The rebound strength to passive movement increased significantly from T_0_ to T_2_ (*p* < 0.01) but not from T_0_ to T_1_ or from T_1_ to T_2_. In the healthy hemisphere, the rebound strengths to tactile stimulation did not differ from those of the controls at any time point whereas the rebound strengths to passive movement were significantly weaker than those of the controls at all time points and remained 67% of the rebound of the controls at T_2_ (*p* < 0.05).

### 3.5. Correlation with Clinical Measures

The rebound strength to tactile stimulation or passive movement did not correlate with the lesion volume at any time point.

#### 3.5.1. Box-and-Block Test of the Impaired Hand

**Figure 3:**
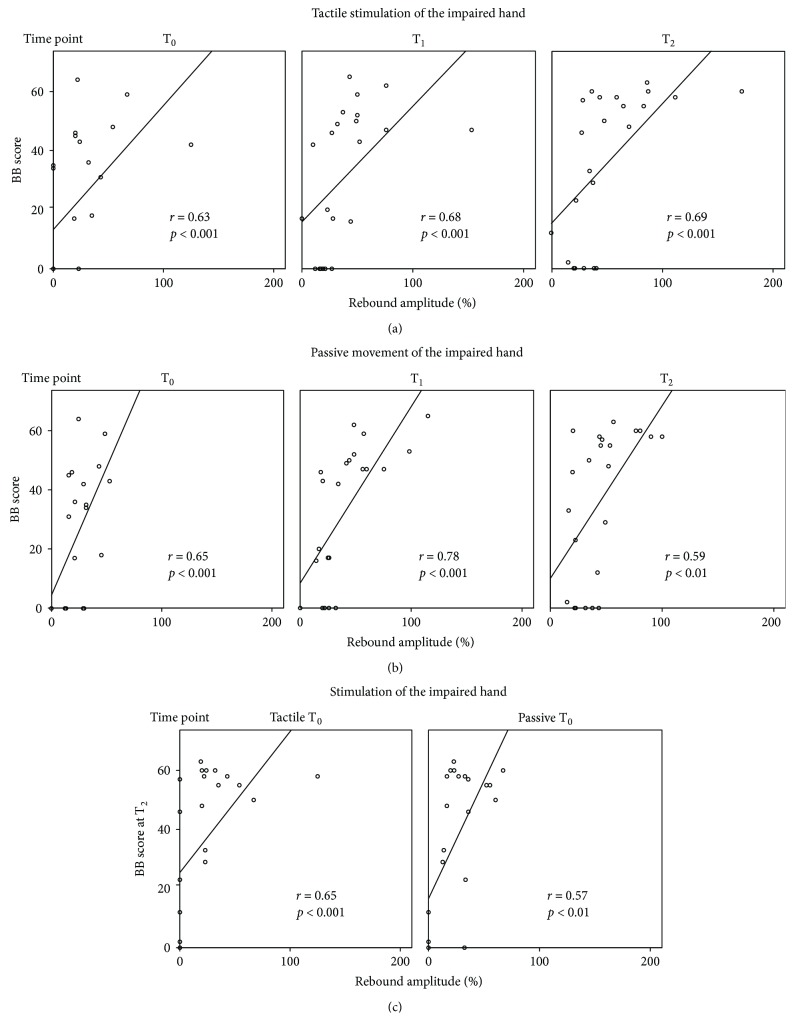
Correlation of the rebound strengths to tactile stimulation and passive movement of the impaired hands with hand motor output. Linear nonparametric correlation of the rebound amplitudes (%) in the affected hemisphere contralateral to (a) tactile stimulation and (b) passive movement of the impaired hands at T_0_–T_2_, and corresponding results of Box-and-Block tests of the impaired hands. T_0_ (1–7 days), T_1_ (1 month), and T_2_ (12 months) after stroke. (c) Correlation of the rebound amplitudes (%) in the affected hemisphere to tactile stimulation and passive movement of the impaired hands at T_0_ with Box-and-Block scores at T_2_.

Figure 3 shows the positive correlation of the AH rebound strength to both stimulus types with BB scores; the stronger the rebound the higher the BB score and the better the motor performance. The Spearman's correlation analysis showed that the rebound strengths to both tactile stimulation and passive movement correlated significantly with BB scores at all time points: to tactile stimulation, *r* = 0.63 and *p* < 0.001 at T_0_, *r* = 0.68 and *p* < 0.001 at T_1_, and *r* = 0.69 and *p* < 0.001 at T_2_ (Figure 3(a)) and to passive movement, *r* = 0.65 and *p* < 0.001 at T_0_; *r* = 0.78 and *p* < 0.001 at T_1_, and *r* = 0.59 and *p* < 0.01 at T_2_ (Figure 3(b)).

Interestingly, the rebound strength at T_0_ correlated significantly with BB scores of the impaired hand at T_2_; the stronger the rebound at T_0_ the better the hand performance at T_2_ (Figure 3(c); *r* = 065, *p* < 0.001 and *r* = 0.57, *p* < 0.01, to tactile stimulation and passive movement, resp.).

#### 3.5.2. Box-and-Block Test of the Healthy Hand

The rebound strength in the unaffected hemisphere to tactile stimulation did not correlate with BB scores of the healthy hand at any time point. The rebound strength in the unaffected hemisphere did not correlate with BB scores of the healthy hand at T_0_ or at T_1_, but a significant correlation was found at T_2_ (*r* = 0.50; *p* < 0.05).

#### 3.5.3. Tactile Sensitivity of the Impaired Hand

The negative correlation of tactile sensitivity with the rebound strength revealed that the better the tactile sensitivity (the thinner the detected von Frey Filament) the stronger the rebound at T_0_ (*r* = −0.57; *p* < 0.01) and at T_1_ (*r* = −0.56; *p* < 0.01) but no significant correlation was found at T_2_.

Tactile sensitivity of the healthy hand did not correlate with the rebound strength in the UH to tactile stimulation at any time point.

## 4. Discussion

In this study, we compared the effect of two types of afferent input, tactile, and proprioceptive stimulation, on the modulation of the 20 Hz rhythm during one-year stroke recovery. The results showed that the rebound strengths to both stimuli were bilaterally diminished in the acute phase. During the first month of recovery, the rebounds increased but after one month, no significant changes were observed.

Temporally similar recovery profiles of the rebounds to both stimuli during the first month suggest that stroke-induced alterations in motor-cortex excitability occur mainly during the first four weeks. This finding confirms and extends the earlier observations indicating a sensitive period for plastic changes during the first weeks after stroke [26–31]. During this rather short period, changes in gene expression and neurotransmission [32–35], altered cortical inhibition [14, 36–42], and structural changes [43–47] enable formation of new networks and reorganization of the sensorimotor cortex.

### 4.1. Rebound Strength in the Acute Phase after Stroke

In the acute phase, the rebound strengths in the affected and unaffected hemispheres to tactile and proprioceptive stimulation were diminished compared to the controls, indicating increased excitability of the motor cortex (Figure 2). This is in line with several earlier studies in both animals and humans, showing hyperexcitability both in the affected and unaffected hemispheres after stroke [14, 18–20, 39, 48–53]. This hyperexcitability—or disinhibition—is suggested to reflect reduced GABA_A_ergic and increased glutamergic activation in the peri-infarct zone and in the contralesional unaffected hemisphere [16, 17, 36, 39, 40, 50–52, 54].

As afferent input also affects motor-cortex inhibition, the observed diminished 20 Hz rebound may result both from decreased cortical inhibition and defective afferent input to the motor cortex [19, 20]. The behavior of the 20 Hz rhythm followed a similar pattern regardless of the stimulus type, corroborating the assumption that defective afferent input alone is not sufficient to explain the decrease in motor-cortex inhibition. This is further supported by the diminished 20 Hz rebound also in the unaffected hemisphere to healthy hand stimulation in the acute phase after stroke. As the afferent input from the healthy hand to the unaffected hemisphere is likely intact, the diminished 20 Hz rebound of the healthy hemisphere probably indicates decreased intracortical inhibition (ICI), whereas in the affected hemisphere, the weaker rebounds likely are due to both decreased ICI and diminished afferent input to the motor cortex. However, the similar reduction in the rebound strengths to both stimuli indicates that in the acute phase, cortical excitability changes modulate the rebound strongly, and this modulation may itself lead to disturbed sensorimotor integration and hence hampered dexterity. This hypothesis is strengthened by the observation that also the healthy hand function was impaired (as compared to the normative values of a healthy population), although no structural lesions were found in the unaffected hemisphere.

### 4.2. Recovery of the Rebound during Follow-Up

The strongest increment of the rebound amplitude occurred from T_0_ to T_1_, whereas no significant increase in the rebound strength was observed from T_1_ to T_2_. Although the changes in the rebound amplitudes to both stimuli followed a rather similar temporal pattern, the rebounds to tactile stimulation reached the level of the controls in both hemispheres during the one-year follow-up, whereas the rebound to passive movement did not. In healthy controls, passive movement has been shown to produce a stronger rebound than electric median nerve stimulation [9] or tactile stimulation [21]. In voluntary movement, the mass of the muscles is known to affect the rebound strength; the greater the mass, the stronger the rebound [55] as a greater mass of moving muscles activate a larger number of sensory afferents.

The weaker recovery of the rebound to passive movement compared with tactile stimulation may imply that in our stroke patients, proprioception did not recover as well as tactile sense. However, this remains speculative as we were not able to precisely define the recovery of proprioception in our patients. Furthermore, anticipation and planning of a forthcoming voluntary movement are known to increase the excitability of the motor cortex, which is reflected in the modulation of the 20 Hz activity but also as the Bereitschaftspotential or readiness field [56, 57]. Although our stimulation did not involve voluntary movement, the timing of the movements was highly predictable and thus at least the healthy subjects could probably anticipate each stimulus, possibly leading to higher rebounds. In addition, our healthy controls might have actively opposed passive movements more than the patients did, particularly since the muscle strength of the patients was diminished. Yet, the significant increase of the rebound strength from the acute phase by one month after stroke was evident. Future studies should be conducted to explore the relationship between recovery of proprioception, muscle strength, and rebound strength.

### 4.3. Rebound Strength and Its Association with Clinical Outcome

In our patients, a stronger rebound (less disinhibition/increased ICI) in the affected hemisphere to both stimulus types was associated with better hand function. Although disinhibition in the acute phase after stroke may be necessary to allow plasticity to a certain extent [3, 5, 58], it is possible that later on a normalization of excitability is a prerequisite for normal or near-normal (sensorimotor integration) and hand functions. Accordingly, the healthy hand function was impaired in the acute phase, concomitantly with a decreased 20 Hz rebound.

Human studies with transcranial magnetic stimulation (TMS) have suggested that reduced short-interval intracortical inhibition (SICI, meaning increased excitability) in an acute stroke enhances afferent input-related long-term potentiation in the motor cortex leading to good motor recovery (measured with modified ranking scale (mRS)) at six months [59, 60]. In stroke patients, a H2O15-PET study revealed bilateral hyperexcitability in the acute phase and a reduced excitability at 31 weeks in both hemispheres in association with better recovery in thumb-to-index finger tapping [61].

By using a paired-pulse transcranial magnetic stimulation (TMS) in stroke patients, motor-cortical disinhibition was found in both hemispheres in the acute phase [39, 40]; increased ICI in the unaffected hemisphere at three months correlated significantly with good hand motor recovery. Accordingly, in patients with poor motor recovery, ICI in the unaffected hemisphere remained high, in line with other studies showing that prolonged hyperexcitation in the unaffected hemisphere would be harmful for recovery after stroke [51, 52]. However, no correlation of increased ICI in the affected hemisphere with clinical recovery was found [39, 40].

Another TMS study by Swayne and colleagues (2008) showed that decreased bilateral ICI did not correlate with hand motor performance in the acute phase after stroke (measured weekly until one month with action research arm test (ARAT) and nine-hole peg test (NHPT)). However, in agreement with our findings, increased ICI in the affected hemisphere at three months correlated strongly with hand motor performance suggesting that new intracortical networks probably were already structured and functioning [47].

Similar findings have been observed in animal studies. In rats, autoradiographics revealed a reduction in GABA_A_ receptor expression in the surroundings of acute photothrombic infarcts [48]. Patch-clamp recordings over the primary motor cortex during acute stroke in mice showed that an occlusion in the middle cerebral artery decreased GABAergic tonic inhibition in conjunction with an activation of N-methyl-D-aspartate (NMDA) receptors in the peri-infarct zone [51]. Taken together, both animal and human studies have indicated decreased ICI in the acute phase after stroke and thereafter increased ICI in association with good motor recovery.

In the present study, the rebound strength in the affected hemisphere to both stimuli correlated significantly with impaired hand motor recovery at all time points indicating that the observed changes in excitability are closely linked to functional recovery. Furthermore, the stronger the rebound in the affected hemisphere in the acute phase the better the hand motor performance at one year, as measured with the Box-and-Block test. Although, the number of patients in our study does not allow to draw direct conclusions, it is a tempting idea that motor outcome after stroke could be predicted by evaluating the initial inhibitory state of the motor cortex with the 20 Hz rebound. This finding could help to develop tools not only for studying alterations in motor-cortex excitability but also for tailoring rehabilitation according to the observed neurophysiological changes and for predicting motor recovery already at acute stage.

## 5. Conclusions

The temporally similar recovery profiles of the 20 Hz rebounds to both tactile stimulation and passive movement indicate that motor-cortex excitability is increased mainly during the first four weeks after stroke, underlining the importance of early and intensive rehabilitation. Furthermore, we found that the rebound strengths in the affected hemisphere to both stimulus types in the acute phase may reflect functionality of sensorimotor integration and predict motor performance in the long run. Importantly, the close connection of afferent input with excitability changes should be paid attention to when planning novel therapeutic interventions. The rebounds to both tactile stimulation and passive movement appear to be robust neurophysiological markers of stroke-induced cortical excitatory changes.

## Figures and Tables

**Table 1:** Clinical details of the patients.

Patient	Gender	Age	NIHSS			Lesion		
			T_0_	T_1_	T_2_	Side	Site	Size (cm^3^)
1	f	68	0	0	0	rh	c	1.78
2	f	59	0	0	0	lh	c	0.24
3	f	60	12	6	4	rh	cs	24.9
4	m	66	4	3	1	rh	cs	71.3
5	m	45	7	2	1	rh	cs	84.2
6	f	58	2	0	0	rh	cs	31.7
7	f	66	5	2	0	rh	cs	4.58
8	m	71	2	1	1	rh	cs	26.7
9	m	75	12	6	2	rh	cs	35.8
10	m	62	3	1	1	rh	cs	21.2
11	m	67	14	10	6	rh	cs	218.5
12	m	47	14	7	5	rh	cs	149.9
13	f	78	7	4	3	rh	cs	55.6
14	m	61	6	4	2	rh	cs	124.8
15	m	49	0	0	0	lh	cs	3.53
16	m	76	4	3	2	lh	cs	2.59
17	f	73	10	4	1	lh	cs	2.84
18	m	68	2	1	1	rh	s	1.36
19	f	59	4	1	0	rh	s	1.95
20	f	75	14	13	4	rh	s	13.0
21	m	64	5	2	1	lh	s	1.46
22	f	74	15	13	6	lh	s	40.0
23	m	74	1	0	0	lh	s	0.48

f: female; m: male; rh: right hemisphere; lh: left hemisphere; c: cortical; cs: cortico-subcortical; s: subcortical; NIHSS: National Institutes of Health Stroke Scale; T_0_: 1–7 days; T_1_: 1 month; T_2_: 12 months from stroke.

**Table 2:** Clinical scores of the patients.

Time	Box-and-Block (mean ± sem)		von Frey (mean ± sem)	
	Impaired hand	Healthy hand	Impaired hand	Healthy hand
T_0_	22 + 4.7^∗∗∗^	45 ± 3	4.56 + 0.22^∗∗^	3.74 ± 0.08
T_1_	32 ± 4.9^∗∗∗^	54 ± 2	4.46 ± 0.23^∗∗^	3.64 ± 0.06
T_2_	36 ± 5.3^∗∗∗^	56 ± 2	4.33 ± 0.24^∗^	3.57 ± 0.04

Box-and-Block: number of blocks replaced in 1 min; tactile sense: von Frey Filaments 1.65–6.65; T_0_: 1–7 days; T_1_: 1 month; T_2_: 12 months from stroke. The significance of the difference between the impaired and healthy hands: ^∗^*p* < 0.05, ^∗∗^*p* < 0.01, and ^∗∗∗^*p* < 0.001.

**Table 3:** Mean strengths (±SEM) of the 20 Hz rebounds in the patients and the controls.

	Patients			Patients			Controls
	AH-impaired			UH-healthy			Contra H
Time	T_0_	T_1_	T_2_	T_0_	T_1_	T_2_	
Rebound to tactile stimulation	22 ± 6.1	41 ± 6.7	50 ± 7.7	47 ± 4.4	66 ± 9.6	73 + 1.1	57 ± 4.9
Rebound to passive movement	24 ± 4.3	39 ± 6.0	44 ± 5.0	48 ± 4.4	59 ± 6.6	65 ± S.5	97 ± 9.3

AH-impaired: affected hemisphere, impaired hand stimulation; UH-healthy: unaffected hemisphere, healthy hand stimulation; T_0_: 1–7 days; T_1_:1 month; T_2_:12 months from stroke; Contra H: hemisphere contralateral to the stimulation. In the controls, contralateral responses in the left and right hemispheres are pooled.
